# Urinary Bisphenol A, F and S Levels and Semen Quality in Young Adult Danish Men

**DOI:** 10.3390/ijerph18041742

**Published:** 2021-02-11

**Authors:** Thea Emily Benson, Anne Gaml-Sørensen, Andreas Ernst, Nis Brix, Karin Sørig Hougaard, Katia Keglberg Hærvig, Jens Peter Ellekilde Bonde, Sandra Søgaard Tøttenborg, Christian H. Lindh, Cecilia Høst Ramlau-Hansen, Gunnar Toft

**Affiliations:** 1 Department of Public Health, Research Unit for Epidemiology, Aarhus University, 8000 Aarhus, Denmark; ags@ph.au.dk (A.G.-S.); aernst@ph.au.dk (A.E.); nis.brix@ph.au.dk (N.B.); chrh@ph.au.dk (C.H.R.-H.); 2 Department of Urology, Regional Hospital of West Jutland, 7500 Holstebro, Denmark; 3 Department of Clinical Genetics, Aarhus University Hospital, 8200 Aarhus, Denmark; 4 Department of Public Health, The Faculty of Health Sciences, University of Copenhagen, 1014 Copenhagen, Denmark; KSH@nfa.dk (K.S.H.); katia.keglberg.haervig@regionh.dk (K.K.H.); Jens.Peter.Ellekilde.Bonde@regionh.dk (J.P.E.B.); 5 National Research Centre for the Working Environment, 2100 Copenhagen, Denmark; 6 Department of Occupational and Environmental Medicine, Bispebjerg and Frederiksberg Hospital, 2400 Copenhagen, Denmark; sandra.soegaard.toettenborg@regionh.dk; 7 Division of Occupational and Environmental Medicine, Lund University, 22363 Lund, Sweden; christian.lindh@med.lu.se; 8 Department of Clinical Medicine, Department of Clinical Epidemiology, Aarhus University, 8200 Aarhus, Denmark; 9 Steno Diabetes Center, Aarhus University Hospital, 8200, Aarhus, Denmark

**Keywords:** bisphenol, endocrine disruptor, epidemiology, male fertility, semen quality

## Abstract

Bisphenol A (BPA) is considered an endocrine disruptor and has been associated with deleterious effects on spermatogenesis and male fertility. Bisphenol F (BPF) and S (BPS) are structurally similar to BPA, but knowledge of their effects on male fertility remains limited. In this cross–sectional study, we investigated the associations between exposure to BPA, BPF, and BPS and semen quality in 556 men 18–20 years of age from the Fetal Programming of Semen Quality (FEPOS) cohort. A urine sample was collected from each participant for determination of BPA, BPF, and BPS concentrations while a semen sample was collected to determine ejaculate volume, sperm concentration, total sperm count, sperm motility, and sperm morphology. Associations between urinary bisphenol levels (continuous and quartile–divided) and semen characteristics were estimated using a negative binomial regression model adjusting for urine creatinine concentration, alcohol intake, smoking status, body mass index (BMI), fever, sexual abstinence time, maternal pre–pregnancy BMI, and first trimester smoking, and highest parental education during first trimester. We found no associations between urinary bisphenol of semen quality in a sample of young men from the general Danish population.

## 1. Introduction

Infertility is a reproductive disorder defined as the inability to achieve pregnancy within twelve months of unprotected and frequent intercourse. The estimated prevalence of infertility among couples is 15%, with up to 50% attributed to male factors [1,2]. Yet, the specific etiological factors of male infertility remain elusive in many cases [3]. It has been proposed that exposure to environmental toxicants, such as endocrine disrupting chemicals, is an important contributor to male infertility [4].

Bisphenol A (BPA) is a ubiquitous endocrine disruptor mainly used in epoxy resins and polycarbonate plastics [5,6]. BPA has become a significant health concern since humans are exposed through ingestion of contaminated food and water, dermal contact, and by inhalation of polluted air [7]. In experimental in vitro and in vivo studies, BPA has exhibited both estrogenic and antiandrogenic properties with deleterious effects on spermatogenesis, thereby affecting semen quality [5,8,9,10,11,12,13]. Among male partners of couples seeking fertility treatment, several studies found that urinary BPA exposure is inversely associated with semen quality, including sperm concentration, total sperm count, and progressive sperm percentage of morphologically normal sperm [14,15,16,17,18]. The influence of BPA exposure on semen quality in the general population has only been investigated in few smaller studies with equivocal findings [19,20,21,22,23,24,25].

Although the estrogenic potency of BPA is still being discussed [26], the endocrine–disrupting properties of BPA exposure has entailed regulation of BPA production, and since 2010, BPA has been banned in several industrialised countries [27]. This led to the development of substances for substitution of BPA, e.g., the structurally similar bisphenol F (BPF) and bisphenol S (BPS). In experimental studies, BPF and BPS have been reported to display similar endocrine-disrupting properties as BPA [28,29,30], but only a single study has investigated the influence of BPS and BPF exposure, in addition to BPA, on semen characteristics in humans [31]. The study observed an inverse association between urinary BPA and BPS concentrations and ejaculate volume, sperm concentration, total sperm count and motility, but no firm conclusions could be drawn in regards to BPF [31].

We aimed to investigate the association between urinary BPA, BPF, and BPS concentrations and semen characteristics among young Danish men from the general population and hypothesised that higher urinary BPA, BPF and BPS concentrations are associated with lower semen quality characteristics.

## 2. Materials and Methods

### 2.1. Study Population and Participants

This cross-sectional study is based on the Fetal Programming of Semen Quality (FEPOS) cohort [32], a sub-study within The Danish National Birth Cohort (DNBC) [33]. Participants were young adult sons born to women enrolled in the DNBC between 1996 and 1999. An information letter was sent to the sons with residency near Copenhagen or Aarhus and with a minimum age of 18 years and 9 months. They were encouraged to participate in the FEPOS cohort if they had descended testicles, and no previous history of sterilisation or chemotherapy. A total of 21,623 men were eligible for participation, and between 2017 and December 2019, 5697 men were consecutively invited to complete an online lifestyle questionnaire, undergo a clinical examination, and provide a semen and a urine sample. Urine samples from a random subset of 556 men were analysed for bisphenols and were included for analysis. A full flowchart of the sampling strategy can be found in Supplementary Material (Figure S1: Flowchart of FEPOS Study Population). Clinical examinations were performed at the Department of Occupational and Environmental Medicine at Bispebjerg and Frederiksberg Hospital in Copenhagen, Denmark and at the Department of Occupational Medicine at Aarhus University Hospital in Aarhus, Denmark.

### 2.2. Urinary Bisphenol A, F and S

Participants provided a single urine sample in a bisphenol-free disposable plastic cup at one of the two hospital clinics. The urine samples were refrigerated at 3–8 °C for a maximum of 12 h and subsequently pipetted into 2 mL test tubes and stored at −80 °C. Urine samples were analysed for BPA, BPF, and BPS by liquid chromatography tandem mass spectrometry (LC-MS/MS; QTRAP 5500, AB Sciex Foster City, CA, USA) [34] at the laboratory at Occupational and Environmental Medicine, Lund University, Sweden. The laboratory in Lund is a reference laboratory for BPA analysis in urine and is part of the Erlangen Round Robin inter-laboratory control program. The laboratory was qualified as an Human Biomonitoring for Europe (HBM4EU) laboratory for the analysis of BPA, BPF, and BPS. Briefly, the urine samples were treated with ß–glucuronidase and incubated at 37 °C for 30 min prior to analysis. Labelled internal standards for each compound were used. All samples were analysed in a random order. The limits of detection (LOD) were 0.2 ng/mL for BPA, and 0.03 ng/mL for BPF and BPS. The coefficient of variance of an included quality control sample were at 8 ng/mL 6% for BPA, at 6 ng/mL 7% for BPF, and at 6 ng/mL 9% for BPS. Creatinine concentrations were determined in the urine samples with an enzymatic method at the Department of Clinical Chemistry, Skåne University Hospital, Lund, Sweden, in order to account for urine dilution.

### 2.3. Semen Characteristics

Participants provided a single semen sample for analysis with a recommended abstinence period of 2–4 days. The samples were collected by masturbation with ejaculation into a plastic container at home or at the clinic. If the sample was collected at home, the participants were requested to keep the semen container at 37 °C by transporting it close to the body. Upon receipt, samples were placed immediately in a heated incubation shaker (37 °C) for approximately 30 min and were processed within a maximum of 1–2 h from collection. Ejaculate volume was assessed by weight (assuming a density of 1 g per millilitre) in a pre-weighed container. Sperm motility was assessed by placing 6 µg of well-mixed semen under a cover slip on a heated (37 °C) microscope slide. In two samples, a minimum of 200 sperm cells were assessed for motility. A mean value was subsequently calculated for each motility group: progressive (PR): spermatozoa moving actively, either linearly or in a large circle, regardless of speed, non–progressive (NP): all other patterns of motility with an absence of progression, e.g., swimming in small circles, the flagellar force hardly displacing the head, or when only a flagellar beat can be observed, immotile (IM): no movement, and total motile (PR+NP) sperm cells [35]. Sperm concentration was determined by manually counting a minimum of 200 sperm cells in two aliquots of a diluted semen sample using an Improved Neubauer Hemocytometer (Paul Marienfield GmbH &Co, Lauda-Königshofen, Germany). Total sperm count was calculated by multiplying the number of spermatozoa (sperm concentration) with ejaculate volume in millilitre. Morphology was determined at the laboratory in Lund, Sweden, by estimating the percentage of normal sperm cells.

Both collection and examination of semen samples followed the World Health Organization (WHO) 2010 standards and requirements [35]. Semen samples were analysed by two trained biomedical laboratory technologists, blinded to exposure status of the participants. The technologists participated in an external quality control programme to ensure compliance with WHO guidelines and ESHIRE External Quality Assessment Scheme. All comparisons were in line with previous studies and further details can be found in Keglberg et al. (2020) [32].

### 2.4. Covariates and Precision Variables

Potential confounding factors were selected a priori based on their anticipated impact on the association between BPA, BPF, and BPS concentration and semen characteristics. Directed Acyclic Graphs (DAGs) were used to illustrate the hypothesised causal structure under study. In addition, precision variables were included in all models to improve precision of the analysis (Figure S2: Directed Acyclic Graph Used for Identification of Confounding Factors and Precision Variables).

Information on smoking status (non–smoker; smoker), alcohol intake (less than once a month; 1–3 times a month; 1–2 times a week; ≥3 times a week), and body mass index (BMI) (<18.5; 18.5–24.9; ≥25 kg/m^2^) was obtained through the self-completed FEPOS questionnaires prior to clinical examination. Information on maternal first trimester smoking status (smoker; non–smoker) and pre-pregnancy BMI (<18.5; 18.5–24.9; ≥25 kg/m^2^) was obtained through a telephone interview completed in the first trimester along with highest parental educational status (high grade professional; low grade professional; skilled or unskilled worker; student or economically inactive).

Precision variables included abstinence time (<2 days; 2–4 days; >4 days), spillage (yes; no), and information on incidences of fever within three months prior to sampling (yes; no) was provided by the participants in a form accompanying the semen sample container. Urine creatinine (mmol/L) was measured and included to control for urine dilution of bisphenol concentrations.

### 2.5. Statistical Analysis

Due to local data regulations, it is not allowed to report values based on less than five observations [36]. Thus, descriptive characteristics for the distribution of urinary bisphenol A, F, and S concentrations and semen characteristics in our study population, are presented using pseudo percentiles, which is the average of the actual percentile and the corresponding two upper and two lower values.

We compared the residuals from standard linear regressions with and without log-transformed semen characteristics to the residuals from a negative binomial regression. The negative binomial regression models were checked by comparing the observed distributions against the model-based distributions from the fitted model using quantile-quantile-plots (QQ-plots). Then, standardised deviance residuals were plotted against model-based predictions. The negative binomial regression model provided the best fit and was used to estimate crude and adjusted ratios (equivalent to percentage change) with 95% confidence intervals (95% CI) between each semen characteristic, and continuous and quartile divided (Q1–Q4) creatinine-adjusted urinary BPA, BPF, and BPS concentrations. The adjusted regression analyses included the abovementioned covariates and precision variables. In addition, the analyses of motility were also adjusted for the number of minutes from sample collection to analysis. Samples for which spillage was reported (*n* = 95) were excluded from the analyses of ejaculate volume and total sperm count according to standard practice [35].

Selection weights measure the inverse probability of participation and were employed to mitigate risk of selection bias due to non-selective participation [37]. Since information on several baseline characteristics was not available for non-participants in the FEPOS cohort, information on maternal characteristics from the baseline questionnaires in the DNBC was used as surrogate markers to estimate the probability of participation. We used a logistic regression analysis with participation (yes/no) as the dependant variable, and the previously mentioned maternal and parental confounders as explanatory variables for participation status. Selection weights were applied to all negative binomial regression models using robust standard errors. Data analyses were performed using STATA version 16.1 (Metrika Consulting AB, Stockholm, Sweden).

## 3. Results

### 3.1. Study Participants

A total of 556 study participants between 18 and 20 years provided semen and urine samples and were included in the analysis. Baseline characteristics are presented in Table 1. The majority had a monthly or weekly alcohol intake (82%), were non–smokers (57%), normal weight (73%), and born to non-smoking (75%) and normal weight (65%) mothers. Distributions of urinary BPA, BPF, and BPS concentrations, and semen characteristics are presented in Table 2 and Table 3.

### 3.2. Urinary Bisphenol Levels

BPA was detected in 95% of urine samples, while urinary BPF and BPS were detected in 92% and 72%, respectively. The median urinary creatinine concentration was 14.31 mmol/mL.

### 3.3. Associations Between Urinary Bisphenol Levels and Semen Characteristics

Adjusted ratios for semen characteristics according to urinary bisphenol concentrations are presented in Table 4, and did not differ notably from the crude ratios. No associations were observed between urinary BPA, BPF and BPS concentrations divided into quartiles and ejaculate volume, sperm concentration, total sperm count or sperm morphology. Modelling BPA, BPF, and BPS as continuous exposures did not reveal other findings. For BPA, the proportion of motile (PR+NP) spermatozoa was 7% (95% CI: 2%; 13%) higher in subjects with urinary concentrations in the third quartile (Q3) compared with BPA concentrations below in the lowest quartile (Q1). For BPF, the proportion of motile (PR+NP) spermatozoa was 6% (95% CI: 1%; 12%) higher for subjects with BPF concentrations in the third quartile (Q3) compared with subjects in the lowest quartile (Q1). Finally, for BPS, subjects in the third quartile (Q3) had 13% (95% CI: −23%; −1%) lower ejaculate volume (mL) compared with subjects in the lowest quartile (Q1).

## 4. Discussion

### 4.1. Key Results

This is the first study to investigate the association between urinary BPA and its analogues, BPF and BPS, and semen characteristics among a sample of young adult men from the general population. Our data did not provide evidence that exposure to BPA, BPF or BPS was associated with semen characteristics. Statistically significant estimates were only observed at intermediate levels, and although non-monotonic dose response associations have previously been observed in relation to BPA exposure [38], no previous studies have shown non-monotonic dose response associations for the specific endpoints studied, and no biological explanation seems likely to substantiate these findings. We therefore consider these results more likely to be attributable to chance findings due to multiple testing.

### 4.2. Strengths and Limitations

The main strength of our study is the large study sample of young men from the general population. Although the participation rate in FEPOS was 19% of which a random set of urine samples were analysed (10%), we were able to take non-participation into account by use of selection weights in the analyses. Moreover, the participants were all young men aged 18–20 years, thus their probability of having attempted to achieve pregnancy and hence be aware of their fertility is considered negligible. All subjects were most likely unaware of their level of bisphenol exposure which further minimises the risk of selection bias [39]. Urine and semen samples were analysed in blind, and routine internal and external quality control was performed revealing high precision and repeatability, hence, diminishing random measurement error.

Due to the cross-sectional design, we relied on a single urine and semen sample from each participant. As the spermatogenic cycle lasts for approximately 90 days, the measured urine levels of bisphenols could have been determined outside sensitive time points [19]. Some studies have found large between-day variability in urinary BPA levels [40,41], but Mahalingaiah et al. (2008) report that a single urine sample may adequately predict long-term exposure [42]. We might also expect a certain degree of intra-individual variation in semen characteristics that cannot be accounted for in the present study. This variation could occur due to random biological variation or the fact that spermatogenesis is more vulnerable to exogenous factors during specific phases, both arguing for repeated sampling as a more reliable indicator for semen quality characteristics. Nevertheless, two studies investigated intra-individual variations of semen characteristics and provided evidence that a single sample may in fact be representative [43,44].

Finally, a major and unique strength in our cross-sectional study was that we were able to control for factors during prenatal life. For instance, maternal smoking and overweight have been suggested to impair gonad development in male offspring affecting semen quality in the long term [45,46,47], and were therefore controlled for in the statistical analyses.

### 4.3. Findings in Relation to Other Studies

Both in vitro and human studies have reported altered semen characteristics in regard to BPA exposure [48,49]. However, findings from human epidemiological studies remain equivocal which could partly be explained by the disparity in exposure levels (Table S1: Overview of Epidemiological Studies Investigating the Associations Between Bisphenols and Semen Characteristics). Furthermore, we still lack evidence on whether BPF and BPS have the same magnitude of potency as BPA.

Similar to our study, Lassen and colleagues performed a cross-sectional study in 2014 including 303 young Danish men attending military service, and examined the association between urinary BPA and semen quality [23]. Although their median BPA concentration was 2.5-fold higher than ours, they only found a statistically inverse association between urinary BPA and percentage of progressive motile sperm cells, while findings for other semen characteristics were not significant [23]. In a Spanish cross-sectional study by Adoamnei et al. including 215 male students aged 18–23, a negative association between urinary BPA and sperm concentration (mill./mL) (ß = −0.04), and total sperm count (mill.) (ß = −0.05) was reported, while other semen parameters were unaffected [19]. Furthermore, no statistically significant associations between urinary BPA concentrations and semen characteristics were reported in an American cross-sectional study by Mendiola et al. (2010) which included 375 male partners of pregnant women with a median urinary BPA concentration of 1.5 ng/mL [24]. This is in line with findings in a more recent cross-sectional study among 105 men recruited from an Italian fertility clinic with remarkably greater median urinary BPA concentration of 0.1 µg/gCr [7].

A wider range of semen quality measures were affected in the cross-sectional study by Meeker et al. (2010) who reported an inverse association between urinary BPA concentrations and sperm concentration, abnormal sperm morphology, and motile sperm among 190 American male partners of couples seeking fertility treatment [15]. Since the design and median urinary BPA concentration is equal to ours (1.30 ng/mL), we suspect that the equivocal findings among the current literature could be explained by other factors than urinary BPA-levels, as discussed below.

A prospective follow-up design may better capture BPA effects in relevant exposure window, since BPA has a short half-life compared to the duration of the spermatogenic cycle of about three months. Goldstone et al. (2015) performed a prospective cohort study including 501 male partners of couples attending fertility treatment. A single urine sample (median BPA = 1.62 ng/mL) and two semen samples were collected at the participants homes. No associations were reported despite separation in time for semen sampling [22]. A similar sampling strategy was used by Pollard et al. (2019) who collected multiple urine samples (GM = 2.5 ng/mL) from 161 fertile men using a home-based kit. No statistically significant associations were observed [25]. Contrarily, Li et al. (2011) performed a prospective cohort study on 218 factory workers from four regions in China by collecting two urine and two semen samples (pre– and post–shift). They found an inverse association between urinary BPA and sperm concentrations, total sperm count, and motility. Differences were only statistically significant when the study population was restricted to those who were highly exposed to BPA through their occupation and had a median urinary BPA concentration of 38.7 µg/gCr compared to factory workers with lower BPA exposure (median urinary BPA of 1.4 µg/gCr) [21].

The effects of BPA exposure may also be explained by the degree of infertility among men being studied. This has been illustrated by the Czechish research group Vitku et al. (2015; 2016) who investigated BPA concentrations in plasma and seminal fluid in men with different degrees of infertility. They found that seminal BPA levels increased with increasing severity of infertility, and that plasma levels of BPA were significantly higher in the groups of slightly and moderately infertile men in comparison with healthy men, and also severely infertile men [17,18]. According to the authors, one possible reason for not finding a clear dose–response relationship between BPA plasma levels and infertility is the role of other factors causing severe infertility, such as genetic or anatomic causes or the result of infections.

The findings are supported by a Greek case-control study reporting stronger association between urinary BPA levels and sperm motility in infertile compared to fertile men [50]. Mantzouki et al. (2019) reached a different conclusion when comparing serum BPA in fertile and infertile men from Greece, since no difference in semen characteristics was reported [51]. However, one must keep in mind that urinary BPA levels do, to a greater extent, reflect the excretion rate of BPA, while measurements of plasma BPA rather reflect its bioavailability. Furthermore, seminal BPA seems to be more appropriate for studying physiology and pathophysiology in the testis [17]. In addition, bisphenol levels are lower in serum than urine samples, and bisphenol F and S are often <LOD in serum, but can be determined more precisely in urine [51]. We therefore chose urine as our source for bisphenol measurements as a proxy of the internal level of exposure.

Only a single study has investigated the association between urinary BPA, and its analogues BPS and BPF, and semen characteristics in male partners of couples seeking fertility treatment. While urinary BPF concentrations were undetectable in most participants, urinary BPA (median = 0.80 ng/mL) and BPS (median = 0.30 ng/mL) concentrations were associated with lower ejaculate volume, sperm concentration, total sperm count and motility. Inverse associations between urinary BPS and semen characteristics were strongest among overweight and obese men after BMI stratification (<25 kg/m^2^ vs. ≥25 kg/m^2^) [31].

An obvious dose–response relationship between the level of bisphenol exposure and impaired semen quality cannot be fully identified in the current literature. However, a tendency towards harmful effects on semen characteristics of BPA exposure in particular seems clear, and thus the analogues, BPF and BPS, must be further investigated in larger epidemiological follow-up studies. Finally, one cannot rule out that publication bias may further contribute to the equivocal findings since fertility studies are cost-intensive and time-consuming.

## 5. Conclusions

In conclusion, we found no association between urinary BPA, BPF, and BPS and semen quality in young adult men from the general population. It seems likely that our exposure levels were too low to find possible effects on semen quality, and hence future studies should preferably have a clearer exposure range including a group of highly exposed subjects.

## Figures and Tables

**Table 1:** Characteristics of 556 Participants from the FEPOS ^1^ Cohort, 2019.

	*n* (%)
Alcohol intake	
Missing	38 (7)
Less than once a month	26 (5)
1–3 times a month	205 (37)
1–2 times a week	248 (44)
≥3 times a week	39 (7)
Smoking status	
Missing	<5 ^2^ (<0.9)
Non–smoker	320 (58)
Smoker	<235 (<42)
Body mass index (kg/m^2^)	
<18.5 (underweight)	45 (8)
18.5–24.9 (normal weight)	407 (74)
>25 (overweight)	102 (18)
Spillage	
Missing	6 (1)
No	455 (82)
Yes	95 (17)
Fever ≤ 90 days prior to sampling	
Missing	63 (11)
No	402 (73)
Yes	91 (16)
Abstinence time (days)	
Missing	<5 ^2^ (<0.9)
<2	193 (34)
2–4	284 (51)
>4	<78 ^2^ (<14)
Maternal smoking status at delivery	
Missing	14 (2.5)
Non–smoker	417 (75)
Smoker	125 (22.5)
Maternal body mass index (kg/m^2^)	
Missing	25 (4.5)
<18.5	53 (9.5)
18.5–24.9	360 (65)
>25	118 (21)
Parental educational level	
Missing	15 (3)
High grade professional	179 (32)
Low grade professional	191 (34)
Skilled or unskilled worker	150 (27)
Student or economically inactive	21 (4)

^1^ Fetal Programming of Semen Quality; ^2^ Due to local data regulations, it is not allowed to report numbers smaller than five, which is why the numbers have been rounded.

**Table 2:** Distribution of Urinary Bisphenol A, F and S Concentrations and Semen Characteristics among Study Participants from the FEPOS ^1^ Cohort, 2019.

	*n*	5th	50th	95th
Bisphenol A (ng/mL)	556	0.22	1.30	9.90
Bisphenol F (ng/mL)	556	<LOD	0.14	2.44
Bisphenol S (ng/mL)	556	<LOD	0.06	1.12
Ejaculate volume (mL) *	455	1.0	2.6	5.4
Sperm concentration (mill./mL)	552	2.1	34.9	130.5
Total sperm count (mill.) *	454	6.3	91.7	390.0
Progressive and non–progressive (%)	542	42.3	71.0	87.0
Morphological normal sperm (%)	541	0.6	6.0	15.0

^1^ Fetal Programming of Semen Quality. All values are reported as pseudo percentiles calculated from the average of five observations. Limit of detection (LOD) is <0.2 ng/mL for bisphenol A, and <0.03 ng/mL for bisphenol F and S. * Participants of which spillage was reported were excluded from ejaculate volume and total sperm count analyses.

**Table 3:** Pseudo Quartile (Q1–Q4) Divided Creatinine-Adjusted Urinary Bisphenol A, F, and S Concentrations among Study Participants from the FEPOS Cohort, 2019.

	BPA	BPF	BPS
Q1 (ng/mL)	<0.68	<0.06	<0.03
Q2 (ng/mL)	0.68–1.30	0.06–0.14	0.03–0.06
Q3 (ng/mL)	1.30–2.74	0.14–0.34	0.06–0.17
Q4 (ng/mL	>2.74	>0.34	>0.017

BPA=Bispehol A; BPF=Bisphenol F and BPS=Bisphenol S.

**Table 4:** Adjusted Ratios of Semen Characteristics According to Urinary Bisphenol A, F, and S Concentrations (ng/mL) among Study Participants (*n* = 556) from the FEPOS Cohort, 2019.

		BPA	BPF	BPS
		Ratios (95% CI)	Ratios (95% CI))	Ratios (95% CI)
Ejaculate volume (mL)	Q1	Reference	Reference	Reference
	Q2	0.97 (0.85; 1.10)	0.91 (0.81; 1.03)	0.97 (0.85; 1.10)
	Q3	1.02 (0.88; 1.17)	0.92 (0.80; 1.05)	**0.87 (0.77; 0.99)**
	Q4	1.04 (0.90; 1.19)	0.96 (0.84; 1.10)	1.00 (0.80; 1.13)
	Continuous	1.00 (0.99; 1.01)	1.00 (0.99; 1.01)	1.03 (0.98; 1.08)
Sperm concentration (mill./mL)	Q1	Reference	Reference	Reference
	Q2	1.10 (0.90; 1.35)	1.11 (0.92; 1.35)	1.06 (0.87; 1.30)
	Q3	0.93 (0.76; 1.15)	1.08 (0.87; 1.34)	0.98 (0.80; 1.19)
	Q4	1.07 (0.86; 1.34)	1.05 (0.85; 1.29)	1.04 (0.85; 1.28)
	Continuous	1.01 (0.99; 1.02)	0.99 (0.98; 1.01)	0.97 (0.88; 1.07)
Total sperm count (mill.)	Q1	Reference	Reference	Reference
	Q2	1.07 (0.86; 1.33)	1.05 (0.84; 1.30)	1.02 (0.81; 1.27)
	Q3	0.93 (0.72; 1.19)	0.97 (0.76; 1.23)	0.91 (0.73; 1.14)
	Q4	1.05 (0.82; 1.35)	0.97 (0.76; 1.23)	1.02 (0.81; 1.30)
	Continuous	1.00 (0.99; 1.01)	0.99 (0.98; 1.01)	1.04 (0.95; 1.13)
Progressive and non–progressive motility (%)	Q1	Reference	Reference	Reference
	Q2	1.05 (0.99; 1.11)	1.04 (0.98; 1.10)	1.03 (0.98; 1.09)
	Q3	**1.07 (1.02; 1.13)**	**1.06 (1.01; 1.12)**	1.02 (0.97; 1.08)
	Q4	1.04 (0.98; 1.11)	1.04 (0.99; 1.11)	1.02 (0.96; 1.08)
	Continuous	1.00 (1.00; 1.01)	1.00 (1.00; 1.01)	1.00 (0.98; 1.02)
Morphological normal sperm (%)	Q1	Reference	Reference	Reference
	Q2	1.03 (0.86; 1.22)	1.09 (0.92; 1.30)	0.98 (0.82; 1.18)
	Q3	0.94 (0.78; 1.13)	1.04 (0.86; 1.27)	1.02 (0.85; 1.21)
	Q4	1.00 (0.82; 1.21)	1.13 (0.93; 1.37)	1.09 (0.90; 1.31)
	Continuous	1.00 (0.99; 1.02)	1.00 (0.99; 1.02)	1.00 (0.93; 1.07)

Estimates marked in bold indicate *p* < 0.05. BPA = Bispehol A; BPF = Bisphenol F and BPS = Bisphenol S.

## Data Availability

The FEPOS study is part of The Danish National Birth Cohort which operates an open access data policy. The DNBC Steering Committee will, however, give priority to projects already launched and agreements made. See https://www.dnbc.dk/access-to-dnbc-data for further information.

## Supplementary Materials

The following are available online at https://www.mdpi.com/1660-4601/18/4/1742/s1, Figure S1: Flowchart of the FEPOS Study Population, Figure S2: Directed Acyclic Graph Used for Identification of Confounding Factors and Precision Variables, Table S1: Overview of Epidemiological Studies Investigating the Association between Bisphenols and Semen Characteristics.
