# Supplementary material for: Urinary Bisphenol A, F and S Levels and Semen Quality in Young Adult Danish Men
Source: Int J Environ Res Public Health. 2021 Feb 11;18(4):1742. doi: 10.3390/ijerph18041742 (PMC7916849; doi:10.3390/ijerph18041742)
Supplement: Supplementary file 1 [file ijerph-18-01742-s001.pdf]

Supplementary Material

Table of Contents

Section A. Supplementary Figures

(I) **Figure S1.** Flowchart of the FEPOS Study Population.

(II) **Figure S2.** Directed Acyclic Graph Used for Identification of Confounding Factors and Precision Variables.

Section B. Supplementary Table

(I) **Table S1.** Overview of Epidemiological Studies Investigating the Association Between Bisphenols and Semen Characteristics.

Figure S1. Flowchart of the FEPOS Study Population.

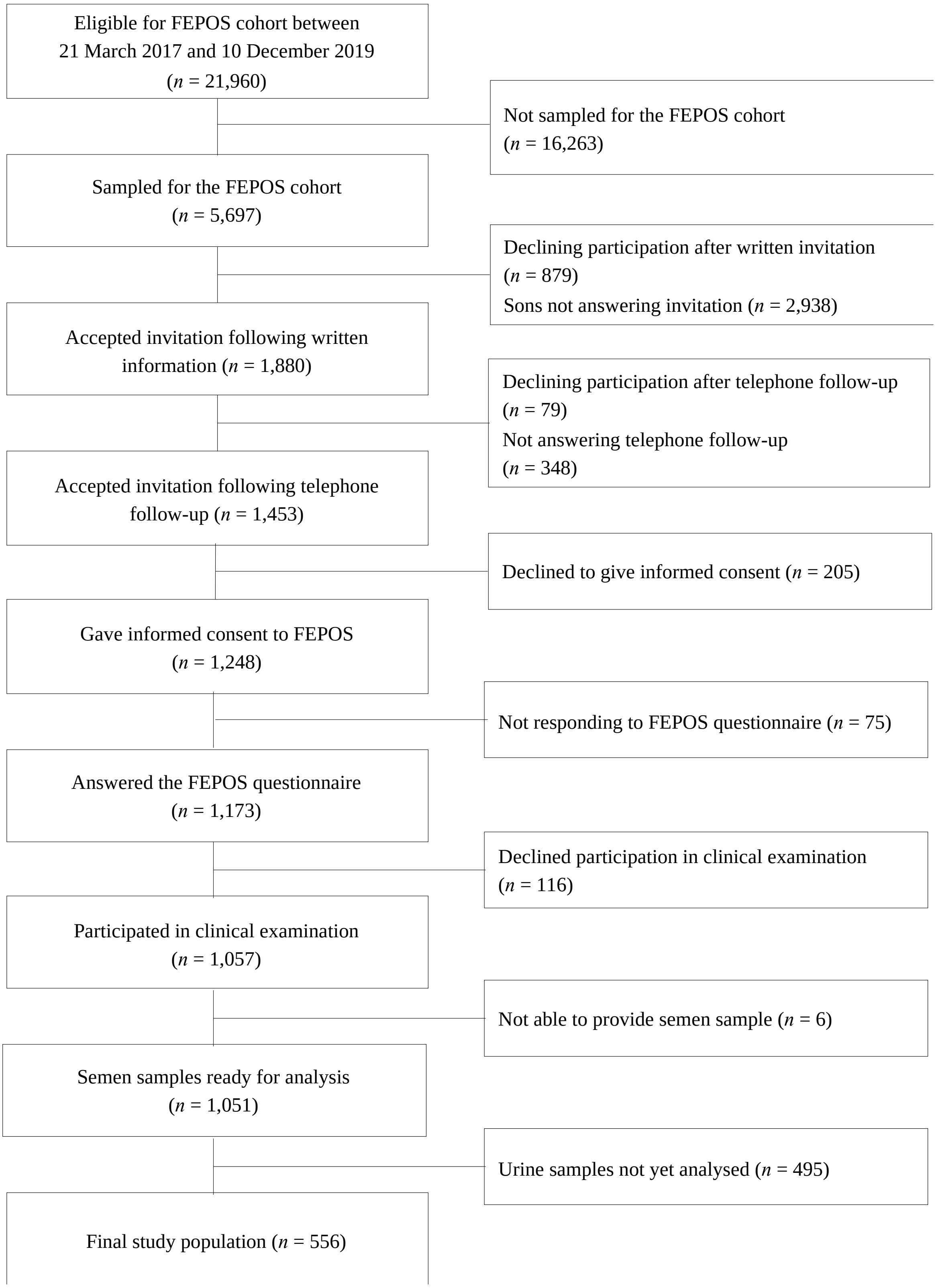

Figure S2. Directed Acyclic Graph Used for Identification of Confounding Factors and Precision Variables.

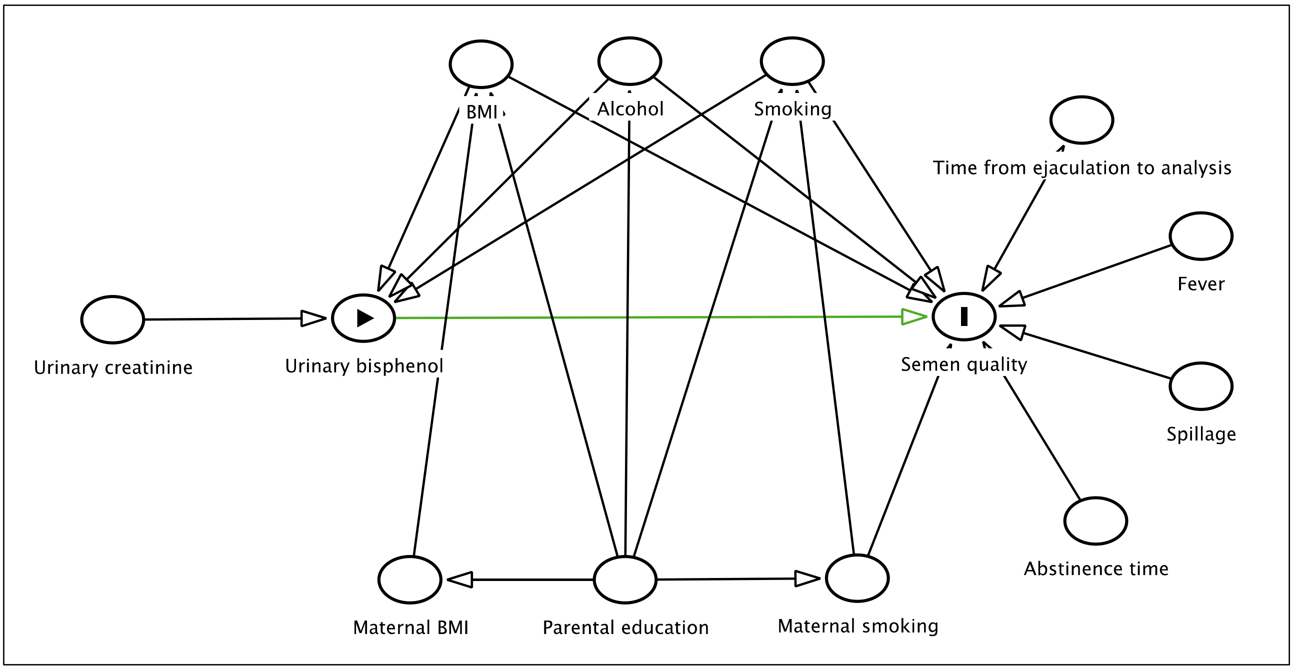

Note: The included precision variables are urinary creatinine levels, time from ejaculation to analysis, spillage, fever and abstinence time, while BMI, alcohol, smoking, maternal BMI, parental education and maternal smoking are considered confounding factors.

Table S1. Overview of Epidemiological Studies Investigating the Association Between Bisphenols and Semen Characteristics.

Study (year)	Study design	Study population	Median urinary bisphenol concentration	Main results
Meeker et al. (2010)	Cross–sectional	Infertile population	BPA: 1.3 ng/mL	Significant association
Mendiola et al. (2010)	Cross–sectional	General population	BPA: 1.5 ng/mL	No significant association
Li et al. (2011)	Cohort study	General population<br>Occupational exposure to BPA	BPA: 1.4 μg/gCr; 38.7 μg/gCr	Significant association
Lassen et al. (2014)	Cross–sectional	General population	BPA: 3.25 ng/mL	No significant association
Goldstone et al. (2015)	Cohort study	General population	BPA: 1.62 ng/mL	No significant association
Vitku et al. (2015)	Cross–sectional	Infertile population	pBPA[a]: 33–47 pg/mL[c]<br>sBPA[b]: 66–179 pg/mL[c]	Significant association
Vitku et al. (2016)	Cross–sectional	Infertile population	pBPA[a]: 0.019–0.072 ng/mL<br>sBPA[b]: 0.075–0.153 ng/mL	Significant association
Adoamnei et al. (2018)	Cross-sectional	General population	BPA: 2.3 ng/mL	No significant association
Omran et al. (2018)	Case–control	General and infertile population	BPA: 24.2 ng/mL; 20.9 ng/mL	Significant association
Ghuyda et al. (2019)	Cross–sectional	Infertile population	BPA=0.80 ng/mL<br>BPS=0.30 ng/mL<br>BPF<LOD	Significant association
Mantzouki et al. (2019)	Case–control	General and infertile population	BPA: 0.19 ng/mL; 0.18 ng/mL	No significant association
Pollard et al. (2019)	Cohort study	General population	BPA: 2.5 ng/mL[d]	No significant association
Caporossi et al. (2020)	Cross–sectional	Infertile population	BPA: 0.1 μg/gCr	No significant association

[a]Plasma BPA
[b]Seminal BPA
[c]Mean
[d]Geometric mean
